# Reply to: A balanced measure shows superior performance of pseudobulk methods in single-cell RNA-sequencing analysis

**DOI:** 10.1038/s41467-022-35520-x

**Published:** 2022-12-22

**Authors:** Kip D. Zimmerman, Ciaran Evans, Carl D. Langefeld

**Affiliations:** 1 Center for Precision Medicine, Wake Forest School of Medicine, Winston-Salem, NC USA; 2 Department of Statistical Sciences, Wake Forest University, Winston-Salem, NC USA; 3 Department of Biostatistical Sciences, Wake Forest School of Medicine, Winston-Salem, NC USA

**Keywords:** Bioinformatics, Computational science, Communication and replication, Statistical methods, Computational models

**replying to** Murphy, A. E. & Skene, N. G. *Nature Communications* 10.1038/s41467-022-35519-4 (2022)

The purpose of our publication “A practical solution to pseudoreplication bias in single-cell studies” was to address a need to reduce the number of studies that do not account for within-individual correlation and report astronomically false associations^1^. The issue is a statistical hypothesis testing question, and our perspective is influenced by the theory underlying the analysis of correlated data^2–4^. Both pseudobulk approaches and mixed models account for the within-sample correlation. Murphy and Skene^5^ focus on a minor point in our paper: the relative performance of the pseudobulk approach in comparison to the two-part hurdle mixed model. We concluded that pseudobulk methods “are only slightly underpowered relative to mixed effects models when there is balance in the numbers of cells per individual, [but] not as well powered as mixed effects models when the numbers of cells per individual grow increasingly imbalanced.” While recognizing that pseudobulk approaches are easy to compute and perform well for basic hypotheses, we noted that they are less flexible and slightly conservative. By definition, a statistical test is conservative if the type 1 error rate is less than the nominal level^6^. Mixed models directly model within-individual correlation, are slightly more powerful with unbalanced samples, and test more complicated hypotheses, but are more computationally intensive. We previously recommended the two-part hurdle mixed model because it estimates the proportion of expressing cells as well as the difference in magnitude conditional on expression^7^. Our recommendation was thus not solely based on power and type 1 error rate. Rather, our intent was to provide the community with an immediately actionable method, consistent with robust statistical theory and with great flexibility in modeling a range of hypotheses. Our recommendation, although not the only reasonable approach, is consistent with the established standard approaches of repeated measures and clustered data analysis.

When comparing statistical tests, it is necessary to establish the size of the test (type 1 error rate) before determining the comparative power relative to those tests with appropriate size. Thus, type 1 and type 2 error rates are not treated equally. Neither the Matthew’s Correlation Coefficient (MCC) nor the receiver operating characteristic (ROC) curves use this classic statistical paradigm of maximizing power after first controlling type 1 error. The MCC is simply the correlation coefficient for a 2 × 2 table and is commonly used to summarize the performance of binary classifiers. The MCC can be expressed as:

1 $${MCC}=\frac{{power}-{type}\,1\,{error}\,{rate}}{\sqrt{\frac{1}{\pi \left(1-\,\pi \right)}(\pi \left({type}\,1\,{error}\,{rate}\right)+\left(1-\pi \right){power})(\pi \left(1-{type}\,1\,{error}\,{rate}\right)+(1-\,\pi )(1-{power}))}}$$

where π represents the proportion of hypotheses expected to be rejected under the null. Clearly, if the type 1 error rates are the same, then the test with greater power would have a larger MCC and the MCC agrees with the classic hypothesis testing paradigm. If two tests have different type 1 error rates, the MCC can favor a test which fails to control the type 1 error rate. In fact, as illustrated in Figure 1 of Murphy and Skene’s manuscript, failing to account for the within-sample correlation causes very high type 1 error rates (>0.50) and yet yields high MCC values^5^. Conversely, as observed from Eq. 1, a smaller type 1 error rate can also lead to a larger MCC. On the surface, a smaller type 1 error rate seems desirable. In classical statistical reasoning, such a test should be recalibrated to have the appropriate size in order to (1) understand the expected number of false rejections and apply appropriate multiple comparison methods, and (2) balance type 1 error rate and power relative to the specifics of the application (e.g., clinical trial, scRNA-seq). Importantly, ranking different methods by MCC means we are ranking as a function of the unknown proportion under the alternative hypothesis, power, and type 1 error rate jointly. However, the proportion and power are dependent on the specifics of the two-parameter alternative hypothesis, and the resulting relationship need not be monotonic. Although exceptions may exist, we generally prefer tests with type 1 error as close to the stated nominal level (e.g., 0.05, 0.001) and do not endorse the application of MCC in this context.

Murphy and Skene also use ROC curves to compare the power of different tests at the same type 1 error rate. Unlike the MCC, ROC curves can be interpreted in the classic hypothesis testing framework: a method with a higher AUC tends to be more powerful at any given level of type 1 error and would be preferred if the rejection threshold has a known error rate. However, the threshold for a conservative test to achieve a type 1 error rate of 0.05 may not be known, and this information is hidden by the ROC curve. In the absence of a systematic method for selecting the rejection threshold, we prefer tests with known error.

Importantly, mathematically, the mixed model parameter estimate has variance less than or equal to that of the pseudobulk estimate, with exact equality only if there is an equal number of cells from each individual. As a result, the power of the mixed model test is no smaller than that of the pseudobulk test and is slightly greater in the unbalanced setting. In reality, we never see a perfectly balanced design (though in practice the difference in power is small). Our original simulations and statements are consistent with these theoretical conclusions^1^.

In addition to the limitations of MCC and ROC curves for comparing hypothesis tests, there are several misleading components we would like to address^5^. First, in Supplementary Figure 1 of Murphy and Skene, the two-part hurdle model is missing and adding it would reveal how much closer the two-part hurdle model is to the nominal *p*-value than the pseudobulk methods. Second, what Murphy and Skene state as a “flaw” in our simulation regarding normalization is not a flaw. Specifically, we explained in our Methods section that this workflow was implemented to prevent DESeq2’s normalization method from removing the simulated effects. Contrary to Murphy and Skene^5^, we also note that the pseudobulk test, which has a type 1 error rate less than nominal level, is by definition a conservative test. Lastly, Murphy and Skene rightfully point out that our simulations to obtain type 1 errors for each method were not completed on the exact same datasets. However, this is again a very minor issue as both of our simulations are based on random draws from the identical distributions. The type 1 errors presented in Table 1 of our manuscript are based on 250,000 iterations and are consistent with Murphy and Skene’s results in Supplementary Figure 1 which are based on 20,000 iterations. Thus, our estimates go the right place, but the bound on error of our estimates is markedly smaller than Murphy and Skene’s bound on error.

In conclusion, it is of critical importance to account for the within-individual correlation in scRNA-seq data, which produces grossly inflated false positives when ignored. The two-part hurdle mixed model is one powerful and flexible option which controls type 1 error, is easily implemented with existing software, and is consistent with statistical literature on the analysis of clustered data. While aggregate methods are often a suitable alternative, we do not endorse the use of MCC as the metric for comparing hypothesis testing methods and we do not believe the differences observed by Murphy and Skene are sufficient to warrant ignoring mixed models.

## Reporting summary

Further information on research design is available in the Nature Portfolio Reporting Summary linked to this article.

## Supplementary information

Reporting Summary
